# Aptamer nucleotide analog drug conjugates in the targeting therapy of cancers

**DOI:** 10.3389/fcell.2022.1053984

**Published:** 2022-12-05

**Authors:** Yongshu Li, Jing Zhao, Zhichao Xue, Chiman Tsang, Xiaoting Qiao, Lianhua Dong, Huijie Li, Yi Yang, Bin Yu, Yunhua Gao

**Affiliations:** ^1^ College of Physics and Optoelectronic Engineering, Shenzhen University, Shenzhen, China; ^2^ Shenzhen Institute for Technology Innovation, National Institute of Metrology, Shenzhen, China; ^3^ Center for Advanced Measurement Science, National Institute of Metrology, Beijing, China; ^4^ Scientific Research Center, The Seventh Affiliated Hospital of Sun Yat-Sen University, Shenzhen, China; ^5^ Department of Anatomical and Cellular Pathology, State Key Laboratory of Translational Oncology, The Chinese University of Hong Kong, Hong Kong, China

**Keywords:** targeting therapy, cancer, nucleotide analog drug, aptamer–drug conjugates (ApDCs), aptamer

## Abstract

Aptamers are short single-strand oligonucleotides that can form secondary and tertiary structures, fitting targets with high affinity and specificity. They are so-called “chemical antibodies” and can target specific biomarkers in both diagnostic and therapeutic applications. Systematic evolution of ligands by exponential enrichment (SELEX) is usually used for the enrichment and selection of aptamers, and the targets could be metal ions, small molecules, nucleotides, proteins, cells, or even tissues or organs. Due to the high specificity and distinctive binding affinity of aptamers, aptamer–drug conjugates (ApDCs) have demonstrated their potential role in drug delivery for cancer-targeting therapies. Compared with antibodies which are produced by a cell-based bioreactor, aptamers are chemically synthesized molecules that can be easily conjugated to drugs and modified; however, the conventional ApDCs conjugate the aptamer with an active drug using a linker which may add more concerns to the stability of the ApDC, the drug-releasing efficiency, and the drug-loading capacity. The function of aptamer in conventional ApDC is just as a targeting moiety which could not fully perform the advantages of aptamers. To address these drawbacks, scientists have started using active nucleotide analogs as the cargoes of ApDCs, such as clofarabine, ara-guanosine, gemcitabine, and floxuridine, to replace all or part of the natural nucleotides in aptamer sequences. In turn, these new types of ApDCs, aptamer nucleotide analog drug conjugates, show the strength for targeting efficacy but avoid the complex drug linker designation and improve the synthetic efficiency. More importantly, these classic nucleotide analog drugs have been used for many years, and aptamer nucleotide analog drug conjugates would not increase any unknown druggability risk but improve the target tumor accumulation. In this review, we mainly summarized aptamer-conjugated nucleotide analog drugs in cancer-targeting therapies.

## Introduction

Cancer is a type of disease attributed to uncontrolled cell growth and division and is usually caused by gene mutation, exposure to radiations and chemicals, and pathogenic infections (Gopinath et al., 2010). Now, cancer has become the second leading cause of death just below heart ailments worldwide (Sung et al., 2021). With the advances in surgical operation, precise radiotherapy, chemotherapeutics, biomedicine-led precision medicine, and immunotherapy, the 5-year survival rate has increased a lot after stepping into the 21st century. In ongoing studies specifically focused on the mechanism of oncogenesis and cancer development, more challenges have been found, such as chemo- and radiotherapy resistance and cancer metastasis and recurrence. In addition, patients desire higher life quality and fewer adverse events through prolonged life expectancy and the development of economics.

**FIGURE 1:**
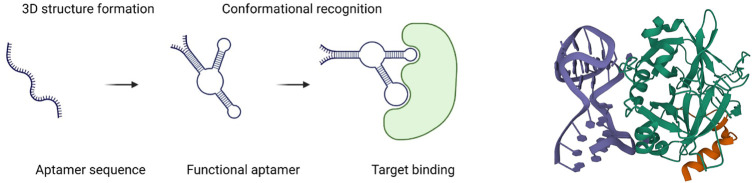
Schematic diagram of an aptamer binding to a target (left) (Sun and Zu, 2015); 3D structure of a 27-mer aptamer and thrombin (right). Aptamer is shown in deep blue, and thrombin, in green (Russo Krauss et al., 2013).

Aptamers consist of single-strand DNA (ssDNA) or RNA and usually include 20–80 nucleotides. The concept of an aptamer was first proposed and proved by Gold’s lab and Szostak’s lab in 1990, independently (Ellington and Szostak, 1990; Tuerk and Gold, 1990; Ku et al., 2015). The ssDNA or RNA can form a secondary structure by hydrogen bond pairing (the Watson–Crick or Hoogsteen type), van der Waals forces, electrostatic interactions, and hydrophobic interactions. Structure determines function, so aptamers, due to their specific 3D structures, can bind different targets with high selectivity and affinity, including carbohydrates, small molecules, peptide toxins, and even whole cells (Yang et al., 2011; McKeague and Derosa, 2012; Mercier et al., 2017) (Figure 1). Aptamers have been widely studied in disease diagnosis and treatment. The aptamer-based biosensors were developed for COVID-19 diagnosis through electrochemical and optical methods (Mandal et al., 2021). Several anti-spike proteins of SARS-CoV-2 were generated to protect against infection (Zhang et al., 2022). In particular, in cancer therapeutics exploration, aptamers can selectively target cancer cells or tissues as drug carriers, showing promising potential in the future for cancer patients and scientists. Recently, Shigdar et al. (2021) described that aptamers are at the cutting edge of cancer therapies, which demonstrated lots of advantages, including enhancing anticancer immunity, developing aptamers targeting hard-to-treat cancers, tracing cancer stem cells, treating nuclear functions, and playing roles as targeting ligands. SELEX (systematic evolution of ligands by exponential enrichment) is the main method to generate aptamers. Generally, it involves positive selection for aptamer enrichment and negative selection to remove the non-specific sequences, just as PCR or transcription are used for sequence amplification. Additionally, there are other non-SELEX ways to generate aptamers developed in the 21st century, which usually mean no PCR amplification step is involved in the protocols. On the other hand, with the accumulation of successful aptamer selection and advances in computer science, *in silico* selection for affinity maturing was explored as well.

As promising candidates in the application of cancer targeted therapy, aptamers have been widely studied in drug delivery due to their high specificity and selectivity (Kim et al., 2021; Yang et al., 2021). Because of their stability and chemically synthesized production, the aptamer–drug conjugate (ApDC) exceeds the capabilities of current antibody–drug conjugates (ADCs). Aptamer acts as a recognition ligand that can target disease sites and/or guide the delivery of multiple therapeutic agents that regulate physiological function by targeting biomarkers. In addition, a DNA aptamer possesses chemical and thermal stability, can be chemically modified easily, is ready for molecular engineering, and can be linked with a variety of therapeutics (Hu et al., 2014; Huang et al., 2014). The RNA aptamer is usually synthesized with chemically modified nucleotides to obtain stability. One nucleotide contains a sugar backbone, a nitrogenous base, and a phosphate group including one to three phosphates as the building block of an aptamer. Moreover, nucleoside analogs have been developed as anticancer drugs for years, such as fluorouracil, gemcitabine, and clofarabine. Therefore, some scientists cleverly combine the aptamer and nucleoside analog drug together for cancer-targeting therapy. Therapeutic nucleotide analogs can replace part of or all nucleotides of aptamers for cancer-targeting therapy but cannot limit the selectivity and affinity (Zhu et al., 2022).

## Aptamer and systematic evolution of ligands by exponential enrichment

**FIGURE 2:**
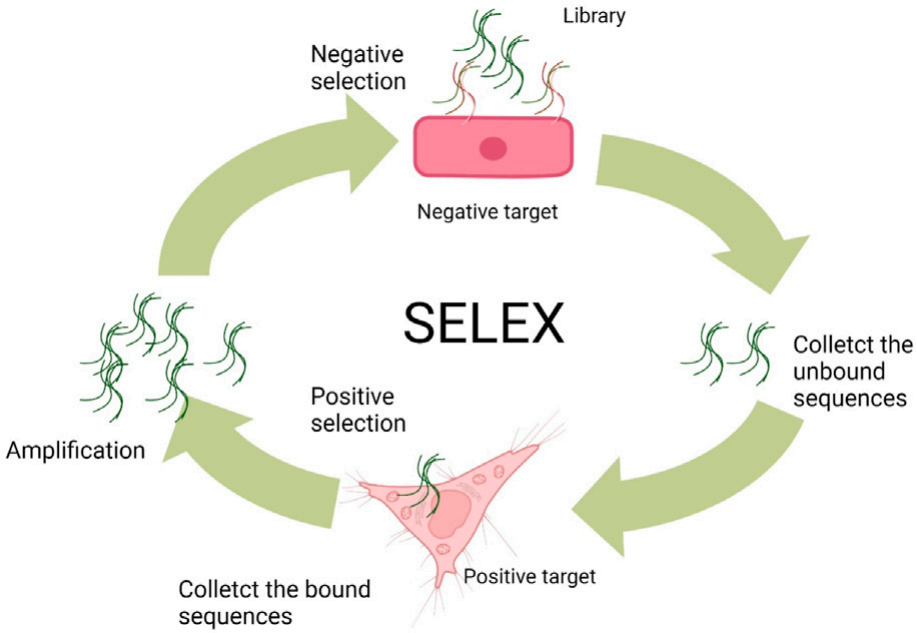
General SELEX procedures (Wu et al., 2017).

Most of the aptamers are selected *via* an *in vitro* and/or *in vivo* selection process known as SELEX from DNA/RNA combinatorial libraries (Ellington and Szostak, 1990; Tuerk and Gold, 1990; Ku et al., 2015). Conventionally, the DNA aptamer SELEX protocols involve three steps. At the beginning, the ssDNA library includes random sequences (A/T/C/G) of 25–45 nucleotides flanked by two primer regions at the 5′ and 3′ terminals, respectively. Principally, a typical library can involve as many as 10^15^–10^27^ random sequences, but only approximately 100 nmol (10^15^) sequences are usually used per SELEX, limited by the synthesized limitation and cost consideration. Second, the library needs to be incubated with the negative (counter) target to remove unspecific binding of ssDNA sequences and then interacted with the positive target for the enrichment of binding sequences with specificity to the target. At the third step, the sequences (aptamers) specifically binding to the positive target would be amplified by polymerase chain reaction (PCR) and then denatured to ssDNA for the next SELEX cycle. As to the RNA aptamer SELEX, the RNA sequence library is produced by transcription from a DNA template containing an additional transcription promoter compared to ssDNA libraries. In the next step, the RNA sequences with affinity to targets are first collected as templates for reverse transcription, and then, the DNA products need to be amplified by PCR. Sequentially, the amplified DNA will be used as templates to transcript RNA products for the next round of SELEX (Figure 2). After obtaining the aptamer pools with specific targeting affinity, the DNA sequencing needs to be performed for picking out the aptamer candidate sequences. These sequences can be synthesized and compared with the affinity and the specificity to target. Usually, the best in class would take part in the following application.

Over the past three decades, SELEX methods have been widely explored by scientists around the world. As a result, a variety of aptamer selection methods have been developed, such as complex target SELEX (Shamah et al., 2008), affinity chromatography SELEX (Liu and Stormo, 2005), capillary electrophoresis SELEX (Mendonsa and Bowser, 2004), genomic SELEX (Kim et al., 2007), monoclonal surface display SELEX (MSD-SELEX) (Zhu et al., 2014), and cell-SELEX (Sefah et al., 2010). No matter how long the names of SELEX have been defined, they can still be divided into three main kinds of SELEX based on the target of selection, namely, protein SELEX, cell-SELEX, and *in vivo* SELEX.

### Protein systematic evolution of ligands by exponential enrichment

Protein SELEX uses purified proteins as the target for generating highly specific aptamers, and the protein is usually a well-identified biomarker. Before SELEX, the targeting protein needs to be fixed to a solid phase, such as magnetic beads or plates, for aptamer binding and non-binding sequence removal. Protein SELEX demonstrates several advantages. For example, it is easy to manipulate without any biohazard component; the binding process happens in the simple solution environment, leading to high success efficiency. The PD-L1-targeting antagonistic DNA aptamer was developed by protein SELEX (Lai et al., 2016). On the other hand, the purified protein may lose its natural 3D conformation under physiological conditions, and the *E. coli*-expressed protein may lack proper post-translational modifications, such as glycosylation modification. In particular, for cell membrane proteins that usually contain a hydrophobic transmembrane domain, they are hard to refine and cannot maintain a suitable fold conformation in a simple solution buffer. The mentioned drawbacks may result in failure upon actual application. Therefore, protein SELEX is usually used in the aptamer selection to target small and simple proteins or a specific domain of a complex protein with high molecular weight.

### Cell systematic evolution of ligands by exponential enrichment

In order to avoid many of the disadvantages of protein SELEX and improve the targeting efficacy for cell surface proteins, cell-SELEX was developed by Shangguan et al. in 2006 (Shangguan et al., 2008; Sefah et al., 2010). In cell-SELEX, the aptamer library would not be incubated with a purified protein but with a particular type of targeting cell line, and then the counter selection would be performed with another control cell line. In aptamer targeting cancer research, it is usually cancer cells and the corresponding normal non-cancerous cells that are the positive selection and negative selection samples, respectively. The selected aptamers can recognize the targeted cell surface proteins in a natural circumstance which would be helpful for new biomarker discovery, application in diagnosis, and drug delivery (Bing et al., 2015). This method may also facilitate the development of highly specific cancer-targeting aptamer probes for internalizing receptors, such as nucleolin and HSP90, which are only displayed as cell surface proteins in cancer cells (Weidle et al., 2011). Eppc6, a DNA aptamer that recognizes EpCAM-positive prostate cancer, was developed by cell-SELEX for *in vitro* study and *in vivo* MR imaging (Zhong et al., 2021).

### *In vivo* systematic evolution of ligands by exponential enrichment

In *in vivo* SELEX, the library is systemically vein injected into a living animal and allowed to circulate through the whole organism. Since there are plenty of RNases existing in animal blood, the random library of 2′-fluoropyrimidine-modified RNA or DNA aptamers is generally used. During the period of aptamer circulation *in vivo*, the aptamers can be distributed throughout the body, and certain aptamer sequences will be accumulated in desirable organs or tissues, while other aptamer sequences would be excluded from the kidney or accumulated in other tissues. After the collection of target organs/tissues, RNA or DNA can be extracted and served as templates for reverse transcription or PCR. The amplified RNA or DNA can be further selected for specific binding to target organs/tissues in the next round of *in vivo* SELEX. After rounds of SELEX, the aptamer sequences will be identified by high-throughput sequencing. A DNA aptamer was developed for bone targeting in the prostate cancer bone metastasis model by *in vivo* SELEX (Chen et al., 2019). In *in vivo* SELEX, it does not only generate aptamers useful for targeting cancer cells, but it can also select aptamers for researching special morphologically or structurally complex healthy tissues. For example, a modified RNA aptamer was selected for penetrating the brain–blood barrier by *in vivo* SELEX (Cheng et al., 2013).

## Non-systematic evolution of ligands by exponential enrichment methods

**FIGURE 3:**
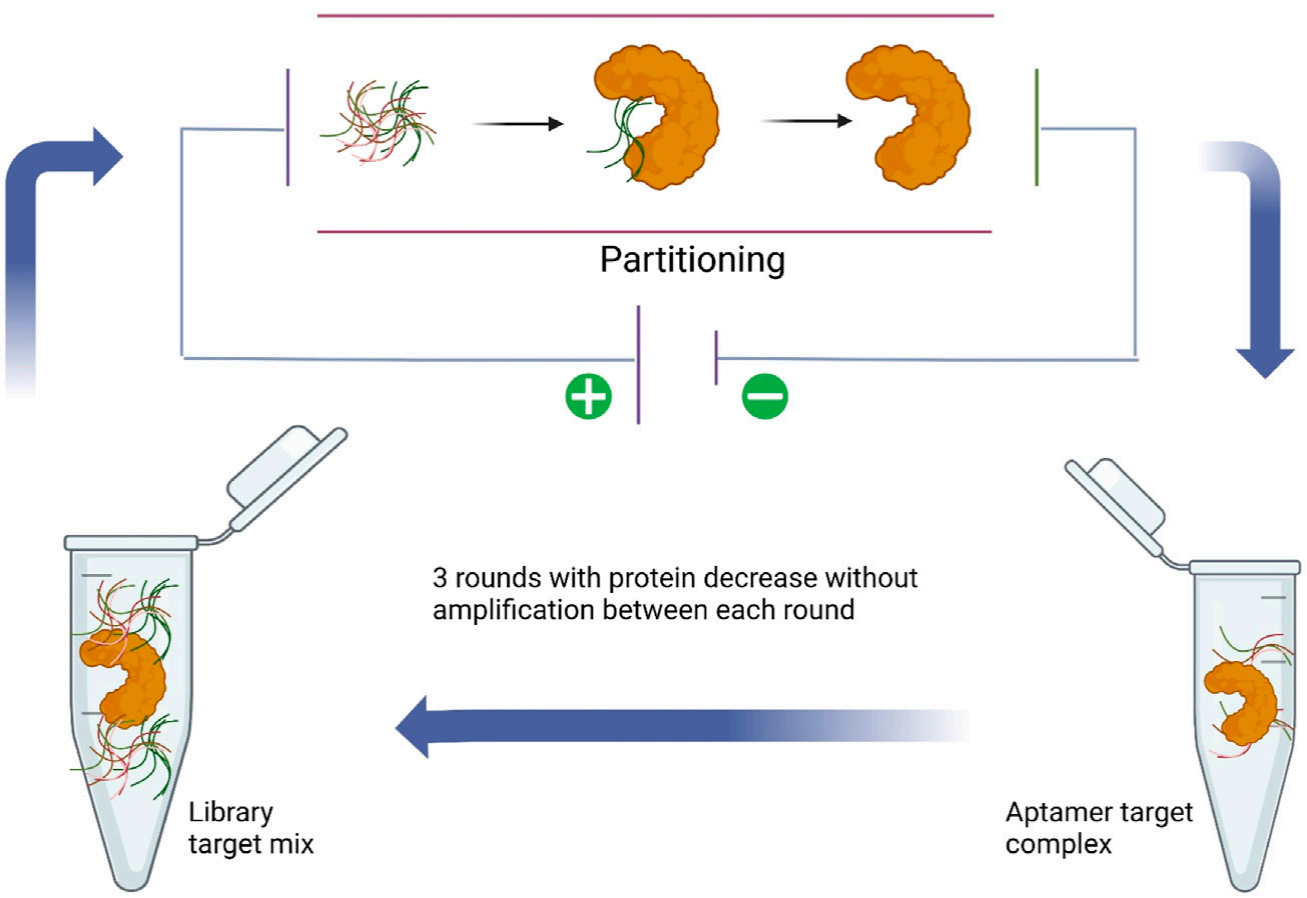
NECEEM-based non-SELEX method (Lisi et al., 2018).

Due to the highly abundant target-non-bound DNA sequences that existed and compared to rare wanted target-bound DNA aptamer sequences, conventional SELEX methods usually need more than 10 rounds of selection, which are time and reagent consumable (Berezovski et al., 2006a). In addition, repeat PCR may accumulate mutations and preferences for different nucleotides because of the polymerase. The non-SELEX methods may avoid these disadvantages of traditional SELEX. Thus, scientists from Canada have developed a non-SELEX aptamer selection method based on electrophoresis first for h-RAS aptamer selection in 2006 (Berezovski et al., 2006b). This non-equilibrium capillary electrophoresis of equilibrium mixtures (NECEEM) used the electrophoresis theory to separate the target protein complex from free DNA sequences, and in this way, only three rounds of NECEEM were used and no PCR amplification was involved (Figure 3). On the other hand, it also provides a new protocol for small-molecule libraries that cannot be PCR-amplified and thus are not suitable for SELEX. Just 4 years later, a variety of non-SELEX conditions were systematically optimized, including the type, concentration, and pH value of the run buffer. Several aptamers were successfully selected for targeting Cdc42-GTP, MRCKa, or PAK1, respectively (Tok et al., 2010). In another research, a total of 10 different ssDNA aptamers were demonstrated to have specific affinity to lipopolysaccharide (LPS) in the nanomolar range by the NECEEM-based non-SELEX method (Kim et al., 2012). The Singapore team also used the NECEEM-based non-SELEX method to generate aptamers targeting bovine catalase with an affinity of around 0.237 µM (Ashley et al., 2012). A mathematical model was developed to analyze the levels of enrichment in non-SELEX selection in a variety of conditions involving different protein concentrations and partitioning (Yu and Yu, 2014). A research team in Italy used the non-SELEX approach based on the capillary electrophoresis partitioning technique to isolate DNA aptamers targeting tau protein, the essential biomarker of Alzheimer’s disease (AD) (Lisi et al., 2018). The final aptamer was calculated with affinity for τ-441, τ-381, τ-352, and τ-383 isoforms at 28, 3.2, 6.3, and 22 nM, respectively. In 2019, scientists from Japan have established a novel approach named “competitive non-SELEX” (and termed “SELCOS” (systematic evolution of ligands by competitive selection)), and they obtained high affinity aptamers to H1N1 (82 pM) and H3N2 (88 pM) (Kushwaha et al., 2019). In addition, they used the H1N1 aptamer to construct an aptasensor for the detection of influenza virus subtypes *via* the electrochemical method. Centrifugation-based partitioning was developed for bacteria-specific aptamer selection by researchers from the Republic of Korea (Kim et al., 2020). A research group from India used the bead-based non-SELEX method to generate an anti-beta-casomorphin-7 (BCM-7) peptide aptamer for the detection of BCM-7 in a human urine sample (Parashar et al., 2022).

**FIGURE 4:**
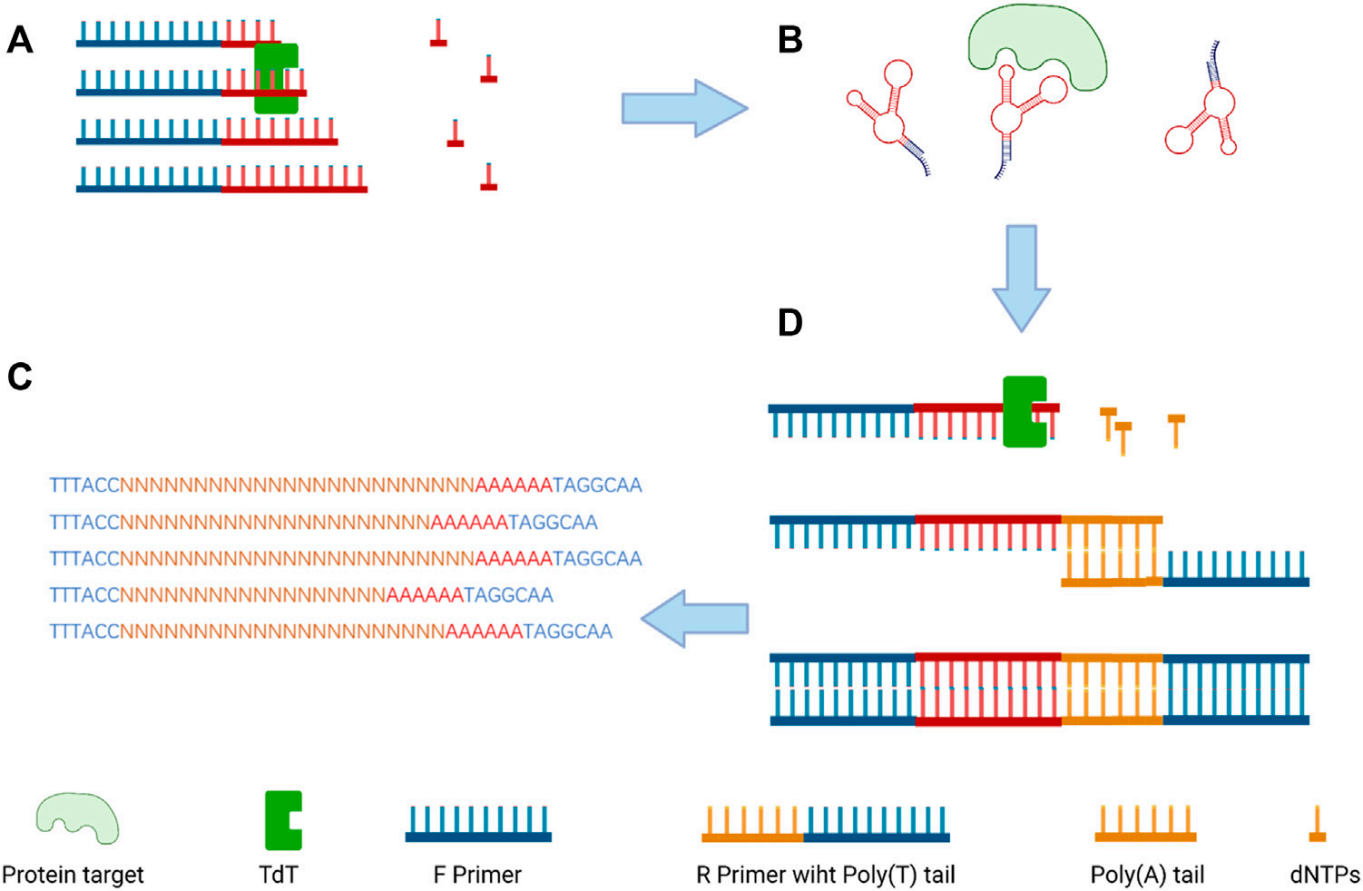
Overview of the selection size-dependent single-round selection of protein-binding polynucleotide using vs-DNA libraries; **(A)** the synthesis of a random ssDNA library *via* TdT; **(B)** the separation of the protein–ssDNA complex; **(C)** the addition of poly-A tail by qPCR amplification to dsDNA; and **(D)** next-generation sequencing (NGS) to analyze the sequences of the candidate aptamer (Ashley et al., 2021).

The terminal deoxynucleotidyl transferase (TDT) enzyme plays an integral part in inducing genetic variation in the variable region of the DNA *via* inserting random nucleotides into the V, D, and J exon regions for recombination of both B and T cells which result in huge diversity of immunoglobulins and T-cell receptors (Sarac and Hollenstein, 2019). As a unique DNA polymerase, TDT can catalyze the stepwise addition of random nucleotides independent of a DNA template (Motea and Berdis, 2010). By taking advantage of TDT, a scientist from Denmark imparts protein antigen-binding properties into nucleic acids to produce a polynucleotide aptamer library (Ashley et al., 2021). Then, the library was incubated with target proteins (thrombin and lactoferrin), and EMSA (electrophoretic mobility shift assays) was used for separating aptamers and the target protein complex for further next-generation sequencing (Figure 4). Through the development of this new non-evolution/non-SELEX screening method, several aptamers were confirmed as specifically binding to thrombin or lactoferrin by SPR (surface plasmon resonance) analysis.

## Aptamer research in cancer imaging and therapy

### Advantageous properties of aptamer for cancer therapy

**FIGURE 5:**
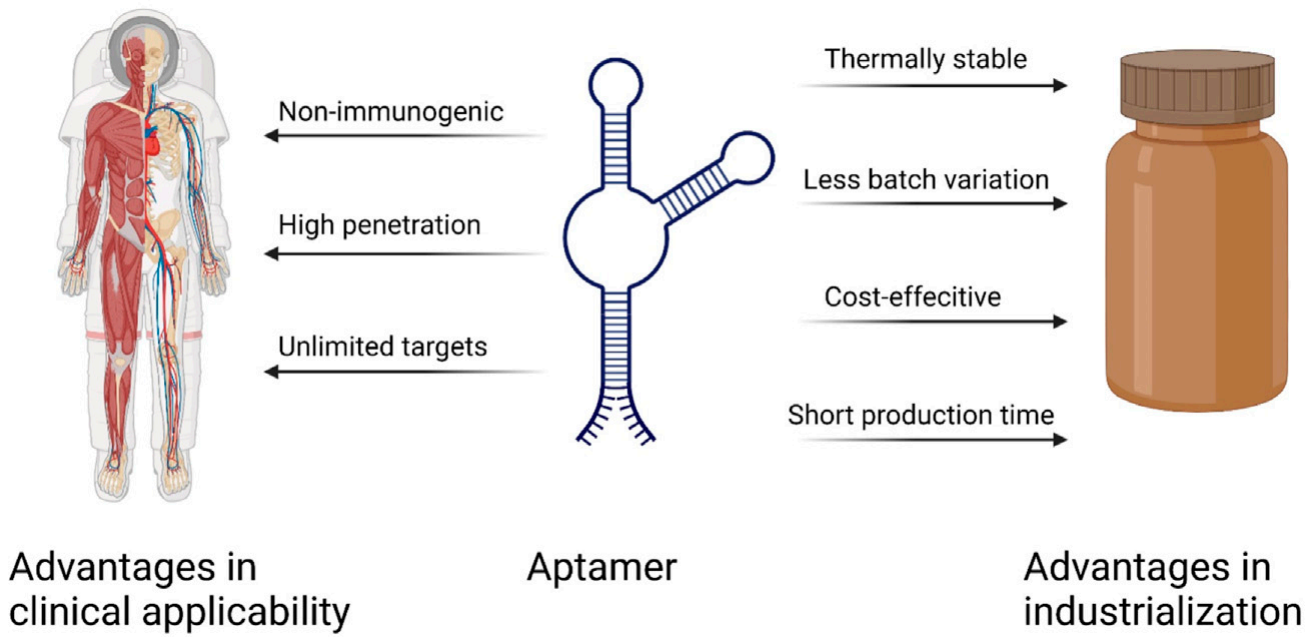
Important advantages of aptamers over antibodies (Sun and Zu, 2015).

There are many advantages to using aptamers as therapeutic agents for targeting cancer therapy compared with other conventional biomolecules such as monoclonal antibodies or cytotoxic drugs(Figure 5). As chemically synthesized biomolecules, aptamer production is easier to scale up during GMP production with limited batch-to-batch variation compared to antibody production. On the other hand, aptamers have high specificity just like antibodies, so they are also called chemical antibodies and have more targeting efficiency than small molecules. In the same way, aptamers can sustain long-term storage in a range of temperatures and solutions. As DNA/RNA drugs, aptamers have lower immunogenicity than proteins (Li et al., 2016). Considering the anti-VEGF pegylated aptamer as an example, it displays low to no immunogenicity in preclinical studies even when the dosage is increased 1,000-fold when comparing the dosage in animal and human therapeutic applications (Rozenblum et al., 2016). The antibody–drug conjugate (ADC) is a well-known missile for cancer therapy, and 11 ADCs have been approved on the market by the FDA (Tong et al., 2021). As to the aptamer–drug conjugate (ApDC), it has similar specificity and affinity, but it is much more convenient to link with other molecules of interest (Keefe et al., 2010). Moreover, compared to the antibody (about 130 kDa), the aptamer has a smaller size (usually between 5 and 15 kDa), which enables high penetration into tissues or cells in solid tumors (Lakhin et al., 2013). Finally, in comparison to the retarded cellular internalization of antibodies with a higher molecular weight, aptamers are usually ready for up-taking by cells through various kinds of mechanisms, and the most common ways are endocytosis and micropinocytosis (Yoon and Rossi, 2018). However, there are still some drawbacks that limit the clinical use of aptamers, such as the aptamer degradation by nuclease in serum, the aptamer excretion from the bloodstream by renal filtration, and the difficulty of interacting with intracellular targets due to the lack of endosomal escape (Lakhin et al., 2013). To address these issues, the modified nucleotide blocks can facilitate the aptamer nuclease resistance, the PEG (polyethylene glycol) conjugates can improve the aptamer circulation in the bloodstream, and most aptamers are generated for targeting cell-surface biomarkers (Rozenblum et al., 2016).

### Aptamer-based therapeutics in current cancer research and clinical study

Totally, 53 clinical trials of aptamer can be searched in NIH (clinicaltrials.gov) until October 2022; eight of them are related to different types of cancer, including CD30-positive lymphomas and solid malignancy imaging, preventing lung cancer in former smokers, acute myeloid leukemia treatment, targeting breast cancer, proteomic biomarker discovery in hepatocellular carcinoma, PET scan imaging in colorectal cancer, and molecular biosensors for the detection of bladder cancer(Table 1). With dramatic progress in aptamer research in cancers, a total of 4,394 results could be found in PubMed until November 2022, and over 500 papers have been published since 2022. More and more researchers have been drawn to aptamer SELEX and its application in cancer diagnosis, tumor imaging, and targeting therapy.

**TABLE 1:** Aptamer studies in cancer clinical trials.

Study title	Condition	Aptamer	Phase	NCT number
Molecular Biosensors for Detection of Bladder Cancer	Urinary bladder	Aptamer sensors		NCT02957370
	Neoplasms			
The Clinical Application of 68Ga Labeled ssDNA Aptamer Sgc8 in Healthy Volunteers and Colorectal Patients	Colorectal cancer	68Ga-Sgc8	Early phase 1	NCT03385148
IST Neoadjuvant Abraxane in Newly Diagnosed Breast Cancer	Breast cancer		Phase 2	NCT01830244
EYE001 to Treat Retinal Tumors in Patients With Von Hippel-Lindau Syndrome	Hippel–Lindau disease	EYE001	Phase 1	NCT00056199
Identify Proteomic Biomarkers for Outcome Prediction of Lipiodol TACE Treatment	Hepatocellular carcinoma	X-aptamer library		NCT04459468
Iloprost in Preventing Lung Cancer in Former Smokers	Lung carcinoma	Aptamer-based analysis	Phase 1	NCT02237183
Alsertib (MLN8237) and Brentuximab Vedotin for Relapsed/Refractory CD30-Positive Lymphomas and Solid Malignancies	CD30-positive lymphoma	Aptamer-mediated flow cytometric detection	Phase 1	NCT02780011
	CD30-positive solid tumor			
A Study of AS1411 Combined With Cytarabine in the Treatment of Patients With Primary Refractory or Relapsed Acute Myeloid Leukemia	Acute myeloid leukemia	AS1411	Phase 2	NCT01034410

### Aptamers function as specific target antagonists

With highly specific affinity, aptamers can even distinguish the protein isoforms precisely. This leads to the discovery of therapeutic aptamers that target the disease-related isoforms but do not affect the function of the normal isoforms. The first FDA-approved aptamer drug, Macugen, was developed to recognize pro-angiogenic VEGF165a, which is the predominantly expressed isoform of VEGF (vascular endothelial growth factor), leading to reducing blood vessel growth and permeability/leakage in neovascular AMD (age-related macular degeneration) (Lee et al., 2005; Ng et al., 2006; Amadio et al., 2016).

In similar fashion, many other aptamers have also been generated to bind their targets competitively, leading to antagonism or activation of the target signal pathway with cancer therapeutic potential. Under normal physiological conditions, PD-1 acts as an immune checkpoint by interacting with PD-L1 or PD-L2 and plays a critical role in decreasing the immune system through suppression of T-cell function, upregulating regulatory T cells (Treg), and thus reducing autoimmunity and promoting self-tolerance in turn. In diverse forms of the tumor microenvironment, T-cell viability can be suppressed by PD-1, leading to the immune escape of PD-L1-expressing cancer cells (Li et al., 2016). An anti-PD-L1 aptamer was generated by the protein SELEX for blocking the signaling pathway and reversing the immune-suppressed tumor microenvironment, which resulted in tumor growth inhibition in the mouse colorectal cancer model (Lai et al., 2016). Another example is an anti-CL4 aptamer that inhibited triple-negative breast cancer (TNBC) cells and tumor growth by impacting the binding between epidermal growth factor receptor (EGFR) and integrin (Camorani et al., 2017).

### Aptamer function as diagnostic tools and imaging agents

Carcinoembryonic antigen (CEA) is widely recognized as a tumor marker in gastrointestinal cancers, especially in colorectal malignancy. Currently, it is detected by immunological methods based on the recognition of antigen by antibodies, such as enzyme-linked immunosorbent assay (ELISA), although they have some limitations, such as high costs and long waiting times in massive population cancer screening (Conry et al., 2000). To address these situations, Kexin Sun and Junlong Li generated an anti-CEA aptamer that would fold into a guanine quadruplex (G4) structure after binding to the CEA protein (Sun and Li, 2022). In other words, the CEA protein would induce the conformational change of this aptamer from a hairpin structure to a G4 structure. This structure transformation can lead to an increase in the fluorescence intensity of TAMRA, which is now used in CEA detection in a much cheaper and faster way.

^18^F-FDG is the standard radiotracer used for PET in cancer patient management. However, it depends on the glucose consumption of tumor cells which means it would not perform well in cancers with low glucose uptake, such as prostate cancer. In addition, some benign tissue proliferation is highlighted in ^18^F-PET scanning, such as thyroid nodules. Therefore, a ^64^Cu-labeled A10 aptamer was developed for prostate cancer PET scan imaging (Kang et al., 2019), which provides another potential noninvasive imaging method for prostate cancer patients. On the other hand, epidermal growth factor receptor (EGFR) is a theragnostic biomarker in many types of cancer, and its aptamer was conjugated with ^18^F-fluorobenzoyl (FB) azide for PET scan imaging in mouse models. In the *in vivo* animal experiment, this aptamer–^18^F conjugate demonstrated the clinical translational potential in human epidermoid carcinoma, human glioblastoma, and human colorectal carcinoma (Cheng et al., 2019). Protein tyrosine kinase-7 (PTK7) is an important member of the receptor tyrosine kinase superfamily and has been demonstrated to be overexpressed in various types of cancer (Jiang et al., 2012; Jin et al., 2014). Sgc8 is an aptamer-targeting PTK7 which was also conjugated with ^18^F for PET scan imaging (Jacobson et al., 2015). This conjugate showed the ability to distinguish not only the primary subcutaneous colon cancer model but also to indicate the liver metastasis model.

### Aptamers function as active molecule delivery vehicles

Aptamer–drug conjugates (ApDCs) have demonstrated several advantages during years of research. Anti-nucleolin aptamer AS1411 has been conjugated with paclitaxel for targeting ovarian cancer (Li et al., 2017). The aptamer improves paclitaxel solubility in an aqueous solution, which benefits drug formulation and effective use. On the other hand, aptamer enhances the targeting efficacy of paclitaxel against tumor cells, leading to a reduction of side effects on normal cells. Except for linking cytotoxic reagents, aptamers were also used to link siRNA for gene knockdown in target cells. EpCAM aptamer was linked with siRNA of UPF2 (up-frameshift suppressor 2), which inhibited tumor growth and enhanced antitumor T-cell immunity in breast cancer in the mouse model (Zhang et al., 2021). Due to the COVID-19 epidemic outbreak over the world, the use of lipid nanoparticles (LNPs) has been accelerated in mRNA vaccines. The aptamer-linked LNP has long been applied in cancer imaging and therapy for years. For example, Chao Liang and his colleagues have used aptamer-functionalized LNP for delivering CRISPR/Cas9 gene-editing constructs of VEGFA in osteosarcoma that inhibited orthotopic OS malignancy and lung metastasis (Liang et al., 2017).

### The application of aptamers with highly specific and efficient internalization capacity

In order to deliver conjugated drugs or linked nanoparticles, the internalization ability of aptamers plays a vital role *in vivo*. Various factors may influence the internalization of aptamers, such as the charge, size, and variation of the 3D structure. After the aptamer binds to the living cells, there are two main internalization mechanisms involved, RME (receptor-mediated endocytosis) and macropinocytosis (Wan et al., 2019). RME is the standard internalization mechanism for most aptamers, and macropinocytosis has only been certified in the internalization of the nucleolin aptamer, and the nucleolin can shuttle between the nucleus and cytoplasm (Bates et al., 2017). Currently, cell-SELEX is the most popular approach to select internalizing DNA and RNA aptamers. For instance, a cell-internalizing DNA aptamer was generated by cell-SELEX in a model system of inflammatory kidney disease (Ranches et al., 2017). RNase was used to facilitate RNA aptamer SELEX, which can digest the surface-binding aptamers but not the cell-internalizing aptamers (Ray and White, 2017). A nuclease-resistant internalizing RNA aptamer was selected for binding to human epidermal growth factor receptor 2 (HER2) through cell-SELEX (Thiel et al., 2012). Generally, the 2′-fluoro-modified RNA library was incubated with cells, and the high salt buffer was used to remove the surface-binding aptamers. In the following step, the internalized RNA aptamers were recovered using the TRIzol reagent.

## Aptamer-conjugated nucleotide analog drugs for cancer-targeting therapy

**FIGURE 6:**
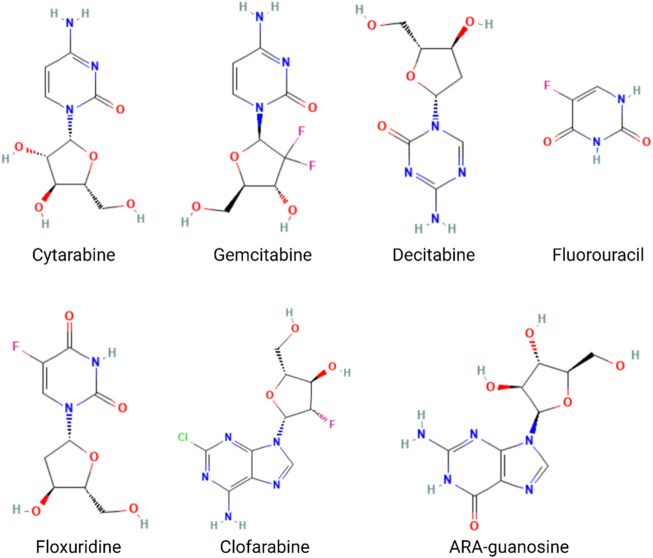
Structures of the nucleotide analog drugs.

The nucleotide analog drugs are not only an important class of antiviral agents commonly used in the therapy of virus infection, including hepatitis B virus (HBV), hepatitis C virus (HCV), human immunodeficiency virus (HIV), varicella-zoster virus (VZV), herpes simplex virus (HSV), cytomegalovirus (CMV), and SARS-CoV-2, but they are also widely used in chemotherapy, as, for example, in the case of cytarabine, gemcitabine, mercaptopurine, azacytidine, cladribine, decitabine, fluorouracil, floxuridine, fludarabine, and nelarabine (Nucleoside Analogues, 2012) (Figure 6). The combination of nucleotide analog drugs and aptamers can minimize the side effects of drugs because of the targeting efficacy of the aptamer. On the other hand, the replacement of some nucleotides in the aptamer sequence by nucleotide analog drugs would usually not impact the aptamer selectivity and affinity to tumor cells. Thus, some research groups have tried to take advantage of their combination in anticancer strategies. Moreover, aptamer-conjugated nucleotide analog drugs avoid the drawbacks (stability/renal excretion) because the degradation can facilitate drug release, and the renal excretion rate is much lower than that of nucleotide analog drugs.

### 5-Fluorouracil-conjugated aptamers used in targeting cancer therapy

5-Fluorouracil (5FU) is a cytotoxic chemotherapy medicine for cancer treatment that is widely used for the treatment of colorectal, esophageal, stomach, pancreatic, breast, and cervical cancers (Fluorouracil, 2021). As a cream preparation, 5FU is used for actinic keratosis, basal cell carcinoma, and skin warts (Moore, 2009; Pharmacists, 2016). As a pyrimidine analog, 5FU would be converted into 5FdU intracellularly, which can inhibit the activity of thymidylate synthase (TS), in turn inhibiting the transformation from dUMP to dTMP. dTMP is the essential ingredient for DNA synthesis. Thus, 5FU disrupts DNA synthesis and acts as an antineoplastic antimetabolite. However, multiple side effects were reported frequently, such as diarrhea; heartburn; sores in mouth and on lips; black, tarry stools; cough or hoarseness, accompanied by fever or chills; lower back or side pain, accompanied by fever or chills; nausea and vomiting (severe); painful or difficult urination, accompanied by fever or chills; and stomach cramps (Fluorouracil, 2022). To address the side effects, various targeting delivery methods of 5FU were developed, mainly including aptamer-modified nanoparticles that are loaded with 5FU (Zhan et al., 2019; Chen et al., 2020; Sathiyaseelan et al., 2021a) and aptamer–5FU conjugates (Yoon et al., 2017a; Sun et al., 2020; Mahajan et al., 2021a). In this section, we mainly summarized the studies of aptamer–5FU conjugates in cancer therapy.

**FIGURE 7:**
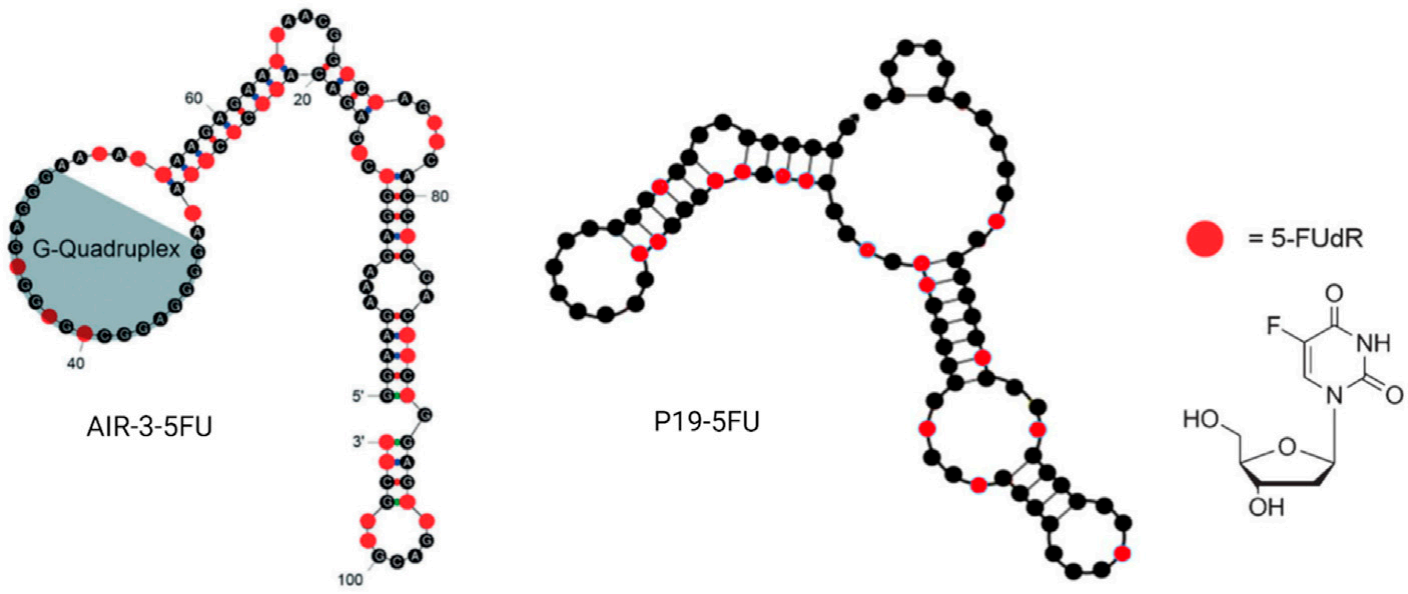
Examples of aptamer–5FU conjugates. AIR-3-5FU aptamer (left); P19-5FU (middle); 5-FUdR chemical structure (right).

As early as 2012, a German group generated an RNA aptamer, AIR-3, targeting human interleukin-6 receptor (hIL-6R) by protein SELEX (Meyer et al., 2012). However, 2 years later, the same group intrinsically embedded 5-fluoro-2′-deoxyuridine (5FdU) into this aptamer for targeted chemotherapy (Kruspe and Hahn, 2014) (Figure 7). Then, 5FdU was conjugated into an aptamer by transcription in the presence of 5FdUTP instead of UTP using the T7 RNA polymerase variant Y639F (Sousa and Padilla, 1995). In an *in vitro* study, significant cell proliferation inhibition was observed in AIR-3-FdU-treated BaF3-hIL-6R cells but not in hIL-6R-negative cells. RNA aptamer SQ-2 was generated by cell-SELEX for targeting alkaline phosphatase placental-like 2 (ALPPL-2) in PDAC cell lines, such as Capan-1 and Panc-1 (Dua et al., 2013). This aptamer was then conjugated with 5FdU at its 3′ terminal by the T7 RNA polymerase variant Y639F-catalyzed transcription and the new developing drug-loaded aptamer named SQ2-5FdU. This SQ2-5FdU showed selective cytotoxicity to ALPPL2-positive Capan-1 PDAC cells but not to ALPPL2-negative HPDE cells (Dua et al., 2015). The RNA aptamer P19 was also generated by cell-SELEX for selectively targeting the pancreatic ductal adenocarcinoma (PDAC) PANC-1 cell line (Yoon et al., 2016). The confocal microscope system was used to evaluate the internalization efficacy of this aptamer on different PDAC cell lines, including PANC-1, AsPC-1, MIA PaCa, and Capan-1 cell lines. Later, the same group conjugated aptamer P19 with gemcitabine and 5FU through transcription (Yoon et al., 2017a) (Figure 7). They also demonstrated selective cell-cycle arrest and cell-proliferating inhibition of P19-conjugated gemcitabine or 5FU in PDAC cell lines but not in normal cells. The Prof. Tan group from Shanghai Jiao Tong University has constructed a bispecific aptamer by DNA polymerase, 5-fluorouracil (5-FU), 10-hydroxycamptothecin, and maleimidocaproyl-valine-citrulline-p-aminobenzoyloxycarbonyl-monomethyl auristatin E (vcMMAE) were conjugated into primers before PCR (Sun et al., 2020). This bispecific aptamer consisted of a targeting aptamer that could bind to the colon cancer cell line HCT116 specifically and another penetrating aptamer (R50) against EGFR-transfected cells. In the end, they proved the constructed bispecific aptamer conjugate can be internalized by cancer cells, specifically showing selective cytotoxicity. Moreover, 5-fluorouracil-chitosan-carbon-quantum-dot-aptamer (5-FU-CS-CQD-Apt) nanoparticles were constructed for targeting MCF-7 breast cancer cells (Sathiyaseelan et al., 2021b). They used the aptamer 5TR1 that was developed in 2009 and targets the cell surface mucin-1 (MUC-1) glycoform (Ferreira et al., 2009; Ray and White, 2010), and the 5FU could be released in a pH-sensitive manner. E07 RNA aptamer was generated by protein SELEX for targeting EGFR, which can block EGF binding to EGFR and inhibit EGF-stimulated EGFR phosphorylation and inhibit A431 cancer cell proliferation in 3D cultures (Li et al., 2011). Scientists from Germany conjugated E07 aptamer 5FU drug units and demonstrated its anticancer capacity in both *in vitro* and *in vivo* studies with an extremely low dosage (Mahajan et al., 2021b).

### Gemcitabine-conjugated aptamers used in targeting cancer therapy

As a nucleoside analog, gemcitabine was originally investigated for its antiviral effects, but now, it has been widely used in various cancer treatments as a nucleotide analog, including but not limited to breast cancer, non-small cell lung cancer, pancreatic cancer, bladder cancer, ovarian cancer, and biliary tract cancer (Leigh Ann Anderson and Kaci Durbin, 2020). *In vivo*, gemcitabine is a prodrug that needs to be activated by deoxycytosine kinase. In turn, it works by replacing the building blocks of nucleotides during DNA synthesis and thus leads to tumor growth arresting at the G1-S border and malignant cell apoptosis. In addition, it was also reported to target the large subunit of ribonucleoside-diphosphate reductase (Kwon et al., 2006; Rosell et al., 2006), thymidylate synthase (Rosell et al., 2004; Huang et al., 2006), and UMP-CMP kinase (Vernejoul et al., 2006; Lam et al., 2007). In particular, in the treatment of NPC, gemcitabine is widely used in chemotherapy combined with 5FU or cisplatin, and gemcitabine can further activate EBV lytic replication, resulting in tumor cell lysis (Stoker et al., 2015). The systemic toxicity to the human body is a huge burden because the gemcitabine can cause cell death to all dividing cells without distinguishing normal cells and cancer cells. This results in many common adverse effects, such as myelosuppression, proteinuria, hematuria, nausea, vomiting, pain, constipation, fever, fatigue, rash, dyspnea, diarrhea, and edema (Company, 2019).

**FIGURE 8:**
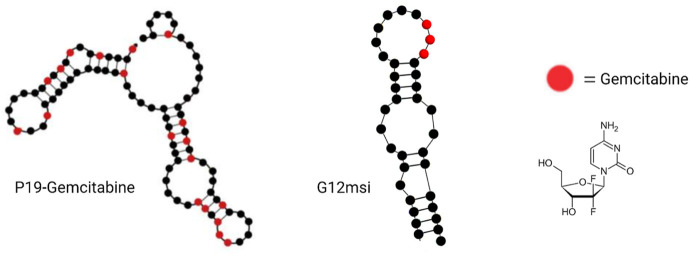
Examples of aptamer–gemcitabine conjugates. P19–gemcitabine aptamer (left); G12msi aptamer (middle); and gemcitabine chemical structure (right).

Aptamer–gemcitabine conjugates can deliver the nucleotide analog drug into cancer cells selectively, maximizing the effectiveness while concurrently minimizing the toxic adverse effects due to the reduced uptake of normal cells (Joshi et al., 2019; Park et al., 2020). As early as 2012, scientists from Duke University designed a gemcitabine polymer and then hybridized it with the anti-EGFR RNA aptamer (E07) by hydrogen bond base pairing, so the aptamer–gemcitabine hybrid can block the EGFR downstream signal pathway and poison the tumor cells with gemcitabine. The cell cytotoxicity of the aptamer–gemcitabine hybrid to the EGFR-positive pancreatic cancer cell line, MiaPaCa-2, has increased significantly but not to the EGFR-negative cell line, HPAF-II (Ray et al., 2012). Later in 2015, gemcitabine was loaded into the AS1411 aptamer surface-decorated nanopolymersome for selectively targeting the nucleolin-overexpressing NSCLC (non-small-cell lung cancer) cell line, A549 (Alibolandi et al., 2016). It enhanced cellular uptake and decreased the IC50 value for A549 cells. Until 2016, researchers from the Beckman Research Institute of City of Hope conjugated gemcitabine into aptamer P19 by RNA polymerase-catalyzed transcription, as P19 is an RNA aptamer targeting PDAC (Yoon et al., 2017b)(Figure 8). The P19–gemcitabine conjugate could be internalized by PANC-1 pancreatic cancer cells, inducing DNA double-strand breaks and inhibiting cell proliferation specifically but not to the non-pancreatic cancer cell line, MCF7 (Yoon et al., 2017b). In 2017, gemcitabine was conjugated in anti-nucleolin DNA aptamer AS1411 during the production process (Park et al., 2018). The 14th nucleotide of AS1411 was replaced by gemcitabine and named APTA-12 sequentially. APTA-12 still showed high specificity and affinity to nucleolin, which is expressed on the cancer cell surface, including Capan-1, MIA PaCa-2, and AsPC-1, but not for the nucleolin-negative cell line, H6c7. The specificity of APTA12 to nucleolin-overexpressed cells and tumor models was also confirmed using PET scan *in vivo* by ^18^F-labeled APTA12 aptamers. In addition, the Capan-1 (pancreatic cancer cell line) tumor mice model was used for testing the therapeutic efficacy of APTA-12, and the results suggest that APTA-12 is a promising anticancer drug in targeting pancreatic cancer therapy. In 2019, scientists from Yeungnam University added doxorubicin (DOX) to APTA-12 to increase the cytotoxicity. Increased cytotoxicity to the TNBC cell line MDA-MB-231 was observed, but only limited and reduced DOX-associated side effects were observed (Joshi et al., 2019). Glypican-3 (GPC3), as a cell surface heparan sulfate proteoglycan, is a valuable therapeutic target and a promising diagnostic biomarker because of its highly specific expression at the plasma membrane of HCC cell lines but not in the normal adult liver tissue (Nakatsura et al., 2003; Yamauchi et al., 2005). In the third decade of the 21st century, a gemcitabine-conjugated anti-glypican-3 (GPC3) aptamer has been developed for targeting hepatocellular carcinoma (HCC) cell lines and xenograft models (Park et al., 2020). This aptamer–gemcitabine conjugate was renamed G12msi which could significantly inhibit the growth of HCC HepG2 cell xenograft models without causing observed toxicity (Figure 8). The ssDNA aptamer PDGC21-T was originally generated for the target gastric cancer cell line BGC-823 by cell-SELEX (Li et al., 2019), but the research group from Houston Methodist Hospital found it can also specifically bind to the TNBC cell line MDA-MB-231. In the following study, they linked three gemcitabine molecules to each PDGC21-T aptamer with a cathepsin B-sensitive linker, so it was named the Apt–cL–triGemcitabine conjugate. Cathepsin B is abundant in the lysosome and can release gemcitabine when the Apt–cL–triGemcitabine conjugate is internalized by cancer cells. In the end, it demonstrated selective cytotoxicity to the TNBC cell line MDA-MB-231 but not to the non-TNBC cell line T47D (Qi et al., 2022). A group from the Yonsei University College of Medicine hypothesized that microscopic residual tumor cells left around the surgical bed could be the reason for future local and systemic recurrences after curative pancreatic resection for pancreatic cancer. In order to improve the efficacy of postoperative chemotherapy and decrease the possibility of recurrences, they developed a pancreatic cancer-specific aptamer-conjugated chemotherapeutic agent-loaded collagen patch for the local release of gemcitabine (Hong et al., 2022). In the PDAC PDX model, the patch demonstrated significant tumor growth inhibition without microscopic evidence suggesting potential toxicity in the liver, lungs, kidneys, and spleen. Also, the TUNEL assay reveals the apoptotic process promotion of PDX tumor.

### Other aptamer-conjugated nucleotide analogs for targeting cancer therapy

Cytarabine is a pyrimidine nucleoside analog that was developed for the treatment of acute non-lymphocytic leukemia, lymphocytic leukemia, and the blast phase of chronic myelocytic leukemia, but it contains some drawbacks, such as rapid liver degradation and significant side effects. To avoid these disadvantages, Fang et al. (2019) from Sun Yat-sen University linked cytarabine to a new GSH-responsive self-assembling small molecular prodrug and then combined it with the Sgc8-BSA aptamer conjugate for improving targeting efficacy. As a tumor-targeting ligand, the Sgc8 aptamer can bind the cancer cell membrane protein, tyrosine kinase-7 (PTK7), which is closely associated with the human leukemic cell line CCRF-CEM cells (CEM cells) (Liu et al., 2015). More recently, the Sgc8 aptamer was reported to be specifically internalized by acute lymphoblastic leukemia (ALL) cells (Abnous et al., 2017). The Sgc8-guided cytarabine-containing complex demonstrated apoptosis induction *in vitro* and antitumor efficacy *in vivo*.

Aptamer A1 was generated by cell-SELEX targeting the NSCLC cell line A549, and this was the first aptamer to demonstrate the capacity to recognize the subcategories of a cancer type in clinical samples (Zhao et al., 2009). The graphene oxide (GO) was conjugated with aptamer A1, and then, the decitabine was loaded onto the surface of the A1-GO conjugate, forming the A1-GO/DAC complex (Lu et al., 2014; Yan et al., 2021). As a chemotherapeutic pyrimidine nucleoside analog, decitabine was used to treat myelodysplastic syndromes (MDS) *via* inhibiting DNA methylation and thus triggering demethylation, leading to the consecutive reactivation of epigenetically silenced tumor suppressor genes *in vitro* and *in vivo* (Hackanson and Daskalakis, 2014). This A1-GO/DAC complex showed specific affinity for A549 cells with pH-sensitive DAC release characteristics.

AS1411 is a DNA aptamer that can work as a targeting ligand and therapeutic agent. It demonstrated tumor cell growth inhibition capacity for a wide range of cancer cell lines (Yazdian-Robati et al., 2020), and it was applied in phase 1/2 clinical trials in the early 21st century. The targeting protein of AS1411 is nucleolin, a protein normally present in the nucleus and cytoplasm but which can translocate to the cell membrane in various types of cancer cells (Chen and Xu, 2016; Jia et al., 2017; Ugrinova et al., 2018). As a pyrimidine analog, floxuridine (FUDR) was used as an antineoplastic agent, usually for the treatment of hepatic metastases from colon cancer *via* continuous hepatic arterial infusion. During intra-arterial manipulation, FUDR is related to a very high rate of serum enzyme and bilirubin elevations, leading to biliary damage frequently and in turn resulting in a secondary sclerosing cholangitis that may further lead to cirrhosis (Information, 2022). Thus, scientists from Korea combined AS1411 and FUDR together by replacing the T of AS1411 with FUDR and naming the modified aptamer F-AS1411 (Tran et al., 2020). The efficacy of F-AS1411 was confirmed with selective affinity and cytotoxicity to CT26 cells (a murine colorectal carcinoma cell). For further improving the delivery and therapeutic effects, they used Phi29 polymerase to amplify poly-F-AS1411 with the c-AS1411 template, and the poly-F-AS1411 can form nanoparticles named NP-F-AS1411. The NP-F-AS1411 demonstrated tumor accumulation and significant therapeutic effect in a mouse model.

Among nucleotide analogs, clofarabine can form base pairing with floxuridine by two hydrogen bonds, mimicking the A:U or A:T base pairs in RNA or DNA aptamer pairs, while gemcitabine and ara-guanosine form another base pair with three hydrogen bonds similar to the G:C pair in aptamers. Thus, Zhu et al. (2022) from Shang Hai Jiao Tong University constructed four drugtamers targeting different cancer cell types. The term drugtamer means all nucleotides in aptamer have been replaced by analog drugs such as clofarabine, ara-guanosine, gemcitabine, or floxuridine, respectively. The four selected aptamers are MUC1 (targeting the mucin-1 protein), Sgc8 (targeting tyrosine kinase-7), SYL3C, and EP116 (targeting the epithelial cell adhesion molecule). After being constructed into drugtamers, the binding affinity (Kd value) of three of them just slightly decreased when compared with the original aptamers, with the following Kd values: for MUC1: 8.7 vs. 11.8, for Sgc8: 9.9 vs. 42.8, and for SYL3C: 29.3 vs. 35.0, while the EP116 drugtamer affinity increased slightly (40.5 vs. 15.7, respectively). All the four drugtamers could induce cell apoptosis *in vitro*, and the therapeutic efficacy of the MUC1 drugtamer was further evaluated *in vivo* in the MCF-1 tumor mouse model. The tumor growth has been inhibited significantly without observed body weight impact. The TUNEL assay and IHC staining of Ki67 and caspase-3 revealed effective inhibition of proliferation and strong apoptosis induction.

## Conclusion and prospects

Aptamers are excellent ligands that can recognize and bind targets, including ions (Guo et al., 2021), small-molecule chemicals (Li and Liu, 2020), RNA G-quadruplexes (Umar et al., 2022), proteins (Mongelard and Bouvet, 2010; Platella et al., 2017), and cells (Chen et al., 2016), with high affinity and specificity. They are recognized as promising targeting ligands in drug delivery, gene therapy, and diagnostics. Through the wide application explored and well-established study results, only one aptamer reached the clinical market nearly 20 years ago, but still, the marketing authorization for Macugen (pegaptanib) has been withdrawn at the request of the marketing authorization holder (Pfizer) in 2011 (Macugen, 2011). Multiple reasons limited its clinical usage, such as rapid filtration and distribution to tissues from the plasma compartment and high nuclease susceptibility, which may lead to the loss of affinity and targetability and result in a low pharmacokinetic profile (Sheikh et al., 2022). Thus, aptamers were chemically modified for nuclease resistance, conjugated with PEG for lower kidney filtration, and associated with nanoparticles for drug and/or gene delivery, but new troubles may emerge due to these new solutions involved, such as affinity decrease and production cost increase. Nucleotide analog drugs have been widely used in various clinical cancer treatments because they share a similar chemical structure with aptamer blocks, nucleotides, or deoxynucleotides at the same time. The long safety and efficacy usage history of nucleotide analog drugs and the structure similarity provide a lot of advantages when conjugating with an aptamer for targeting cancer therapy. They could be conjugated into aptamers easily with very limited structure and specific affinity impact but extremely increased targeting efficacy to avoid adverse cytotoxicity to normal cells, which can result in much lower side effects and higher tumor accumulation. Shigdar et al. (2021) published a review paper on molecular therapy describing how aptamers may be on the cutting edge of cancer therapies. Then, the aptamer nucleotide analog drug conjugates may become the site where the “aptamer knife” can cut into.

## References

[B1] Abnous K. Danesh N. M. Ramezani M. Lavaee P. Jalalian S. H. Yazdian-Robati R. (2017). A novel aptamer-based DNA diamond nanostructure for *in vivo* targeted delivery of epirubicin to cancer cells. RSC Adv. 7 (25), 15181–15188. 10.1039/c6ra28234b

[B2] Alibolandi M. Ramezani M. Abnous K. Hadizadeh F. (2016). AS1411 aptamer-decorated biodegradable polyethylene glycol-poly(lactic-co-glycolic acid) nanopolymersomes for the targeted delivery of gemcitabine to non-small cell lung cancer *in vitro* . J. Pharm. Sci. 105 (5), 1741–1750. 10.1016/j.xphs.2016.02.021 27039356

[B3] Amadio M. Govoni S. Pascale A. (2016). Targeting VEGF in eye neovascularization: What's new?: A comprehensive review on current therapies and oligonucleotide-based interventions under development. Pharmacol. Res. 103, 253–269. 10.1016/j.phrs.2015.11.027 26678602

[B4] Ashley J. Ji K. Li S. F. (2012). Selection of bovine catalase aptamers using non-SELEX. Electrophoresis 33 (17), 2783–2789. 10.1002/elps.201200032 22965726

[B5] Ashley J. Schaap-Johansen A. L. Mohammadniaei M. Naseri M. Marcatili P. Prado M. (2021). Terminal deoxynucleotidyl transferase-mediated formation of protein binding polynucleotides. Nucleic Acids Res. 49 (2), 1065–1074. 10.1093/nar/gkaa1263 33398328 PMC7826267

[B6] Bates P. J. Reyes-Reyes E. M. Malik M. T. Murphy E. M. O'Toole M. G. Trent J. O. (2017). G-quadruplex oligonucleotide AS1411 as a cancer-targeting agent: Uses and mechanisms. Biochim. Biophys. Acta. Gen. Subj. 1861, 1414–1428. 10.1016/j.bbagen.2016.12.015 28007579

[B7] Berezovski M. Musheev M. Drabovich A. Krylov S. N. (2006). Non-SELEX selection of aptamers. J. Am. Chem. Soc. 128 (5), 1410–1411. 10.1021/ja056943j 16448086

[B8] Berezovski M. V. Musheev M. U. Drabovich A. P. Jitkova J. V. Krylov S. N. (2006). Non-SELEX: Selection of aptamers without intermediate amplification of candidate oligonucleotides. Nat. Protoc. 1 (3), 1359–1369. 10.1038/nprot.2006.200 17406423

[B9] Bing T. Shangguan D. Wang Y. (2015). Facile discovery of cell-surface protein targets of cancer cell aptamers. Mol. Cell. Proteomics 14 (10), 2692–2700. 10.1074/mcp.M115.051243 26199357 PMC4597145

[B10] Camorani S. Crescenzi E. Gramanzini M. Fedele M. Zannetti A. Cerchia L. (2017). Aptamer-mediated impairment of EGFR-integrin αvβ3 complex inhibits vasculogenic mimicry and growth of triple-negative breast cancers. Sci. Rep. 7, 46659. 10.1038/srep46659 28425453 PMC5397976

[B11] Chen L. He W. Jiang H. Wu L. Xiong W. Li B. (2019). *In vivo* SELEX of bone targeting aptamer in prostate cancer bone metastasis model. Int. J. Nanomedicine 14, 149–159. 10.2147/IJN.S188003 30613143 PMC6306056

[B12] Chen M. Yu Y. Jiang F. Zhou J. Li Y. Liang C. (2016). Development of cell-SELEX technology and its application in cancer diagnosis and therapy. Int. J. Mol. Sci. 17 (12), E2079. 10.3390/ijms17122079 PMC5187879 27973403

[B13] Chen W. Yang S. Wei X. Yang Z. Liu D. Pu X. (2020). Construction of aptamer-siRNA chimera/PEI/5-FU/Carbon nanotube/collagen membranes for the treatment of peritoneal dissemination of drug-resistant gastric cancer. Adv. Healthc. Mat. 9 (21), e2001153. 10.1002/adhm.202001153 32935949

[B14] Chen Z. Xu X. (2016). Roles of nucleolin. Focus on cancer and anti-cancer therapy. Saudi Med. J. 37 (12), 1312–1318. 10.15537/smj.2016.12.15972 27874146 PMC5303768

[B15] Cheng C. Chen Y. H. Lennox K. A. Behlke M. A. Davidson B. L. (2013). *In vivo* SELEX for identification of brain-penetrating aptamers. Mol. Ther. Nucleic Acids 2, e67. 10.1038/mtna.2012.59 23299833 PMC3564417

[B16] Cheng S. Jacobson O. Zhu G. Chen Z. Liang S. H. Tian R. (2019). PET imaging of EGFR expression using an (18)F-labeled RNA aptamer. Eur. J. Nucl. Med. Mol. Imaging 46 (4), 948–956. 10.1007/s00259-018-4105-1 30069577 PMC6358511

[B17] Company E. L. a. (2019). Gemzar (gemcitabine) prescribing information.

[B18] Conry R. M. Allen K. O. Lee S. Moore S. E. Shaw D. R. LoBuglio A. F. (2000). Human autoantibodies to carcinoembryonic antigen (CEA) induced by a vaccinia-CEA vaccine. Clin. Cancer Res. 6 (1), 34–41. 10656429

[B19] Dua P. Kang H. S. Hong S. M. Tsao M. S. Kim S. Lee D. k. (2013). Alkaline phosphatase ALPPL-2 is a novel pancreatic carcinoma-associated protein. Cancer Res. 73 (6), 1934–1945. 10.1158/0008-5472.CAN-12-3682 23467613

[B20] Dua P. S S. Kim S. Lee D. k. (2015). ALPPL2 aptamer-mediated targeted delivery of 5-fluoro-2'-deoxyuridine to pancreatic cancer. Nucleic Acid. Ther. 25 (4), 180–187. 10.1089/nat.2014.0516 25919296

[B21] Ellington A. D. Szostak J. W. (1990). *In vitro* selection of RNA molecules that bind specific ligands. Nature 346 (6287), 818–822. 10.1038/346818a0 1697402

[B22] Fang Z. Wang X. Sun Y. Fan R. Liu Z. Guo R. (2019). Sgc8 aptamer targeted glutathione-responsive nanoassemblies containing Ara-C prodrug for the treatment of acute lymphoblastic leukemia. Nanoscale 11 (47), 23000–23012. 10.1039/c9nr07391d 31769777

[B23] Ferreira C. S. Cheung M. C. Missailidis S. Bisland S. Gariepy J. (2009). Phototoxic aptamers selectively enter and kill epithelial cancer cells. Nucleic Acids Res. 37 (3), 866–876. 10.1093/nar/gkn967 19103663 PMC2647295

[B24] Fluorouracil. Fluorouracil (5FU). 2021; Available at:https://www.cancerresearchuk.org/about-cancer/cancer-in-general/treatment/cancer-drugs/drugs/fluorouracil .

[B25] Fluorouracil (2022). Fluorouracil side effects. Available at:https://www.drugs.com/sfx/fluorouracil-side-effects.html .

[B26] Gopinath S. C. Wadhwa R. Kumar P. K. (2010). Expression of noncoding vault RNA in human malignant cells and its importance in mitoxantrone resistance. Mol. Cancer Res. 8 (11), 1536–1546. 10.1158/1541-7786.MCR-10-0242 20881010

[B27] Guo W. Zhang C. Ma T. Liu X. Chen Z. Li S. (2021). Advances in aptamer screening and aptasensors' detection of heavy metal ions. J. Nanobiotechnology 19 (1), 166. 10.1186/s12951-021-00914-4 34074287 PMC8171055

[B28] Hackanson B. Daskalakis M. (2014). Recent Results Cancer Res. 201, 269–297. 10.1007/978-3-642-54490-3_18 24756800

[B29] Hong S. S. Lee S. Lee S. H. Kim S. Kim D. Park H. (2022). Anticancer effect of locally applicable aptamer-conjugated gemcitabine-loaded atelocollagen patch in pancreatic cancer patient-derived xenograft models. Cancer Sci. 113 (5), 1752–1762. 10.1111/cas.15318 35243724 PMC9128157

[B30] Hu R. Zhang X. Zhao Z. Zhu G. Chen T. Fu T. (2014). DNA nanoflowers for multiplexed cellular imaging and traceable targeted drug delivery. Angew. Chem. Int. Ed. Engl. 53 (23), 5821–5826. 10.1002/anie.201400323 24753303

[B31] Huang C. L. Yokomise H. Fukushima M. Kinoshita M. (2006). Tailor-made chemotherapy for non-small cell lung cancer patients. Future Oncol. 2 (2), 289–299. 10.2217/14796694.2.2.289 16563096

[B32] Huang F. You M. Chen T. Zhu G. Liang H. Tan W. (2014). Self-assembled hybrid nanoparticles for targeted co-delivery of two drugs into cancer cells. Chem. Commun. 50 (23), 3103–3105. 10.1039/c3cc49003c PMC3973031 24516863

[B33] Information N. C. f. B. (2022). PubChem compound summary for CID 5790. *Floxuridine*. 2022 [cited 2022 June 8]; Available at:https://pubchem.ncbi.nlm.nih.gov/compound/Floxuridine .

[B34] Jacobson O. Weiss I. D. Wang L. Wang Z. Yang X. Dewhurst A. (2015). 18F-Labeled single-stranded DNA aptamer for PET imaging of protein tyrosine kinase-7 expression. J. Nucl. Med. 56 (11), 1780–1785. 10.2967/jnumed.115.160960 26315836 PMC4918919

[B35] Jia W. Yao Z. Zhao J. Guan Q. Gao L. (2017). New perspectives of physiological and pathological functions of nucleolin (NCL). Life Sci. 186, 1–10. 10.1016/j.lfs.2017.07.025 28751161

[B36] Jiang G. Zhang M. Yue B. Yang M. Carter C. Al-Quran S. Z. (2012). PTK7: A new biomarker for immunophenotypic characterization of maturing T cells and T cell acute lymphoblastic leukemia. Leuk. Res. 36 (11), 1347–1353. 10.1016/j.leukres.2012.07.004 22898210 PMC3447106

[B37] Jin J. Ryu H. S. Lee K. B. Jang J. J. (2014). High expression of protein tyrosine kinase 7 significantly associates with invasiveness and poor prognosis in intrahepatic cholangiocarcinoma. PLoS One 9 (2). e90247. 10.1371/journal.pone.0090247 24587299 PMC3938661

[B38] Joshi M. Choi J. S. Park J. W. Doh K. O. (2019). Combination of doxorubicin with gemcitabine-incorporated G-quadruplex aptamer showed synergistic and selective anticancer effect in breast cancer cells. J. Microbiol. Biotechnol. 29 (11), 1799–1805. 10.4014/jmb.1907.07029 31546295

[B39] Kang L. Rosenkrans Z. T. Cai W. Clifton N. J. (2019). Cu-labeled aptamers for tumor-targeted radionuclide delivery. Methods Mol. Biol., 223–231. 10.1007/978-1-4939-9220-1_17 31099007

[B40] Keefe A. D. Pai S. Ellington A. (2010). Aptamers as therapeutics. Nat. Rev. Drug Discov. 9 (7), 537–550. 10.1038/nrd3141 20592747 PMC7097324

[B41] Kim D.-H. Seo J. M. Shin K. J. Yang S. G. (2021). Design and clinical developments of aptamer-drug conjugates for targeted cancer therapy. Biomater. Res. 25 (1), 42. 10.1186/s40824-021-00244-4 34823601 PMC8613924

[B42] Kim H. J. Kwon M. Yu J. (2007). Elucidation of the RNA target of linezolid by using a linezolid-neomycin B heteroconjugate and genomic SELEX. Bioorg. Med. Chem. 15 (24), 7688–7695. 10.1016/j.bmc.2007.08.053 17869523

[B43] Kim H. R. Song M. Y. Chan Kim B. (2020). Rapid isolation of bacteria-specific aptamers with a non-SELEX-based method. Anal. Biochem. 591, 113542. 10.1016/j.ab.2019.113542 31837967

[B44] Kim S. E. Su W. Cho M. Lee Y. Choe W. S. (2012). Harnessing aptamers for electrochemical detection of endotoxin. Anal. Biochem. 424 (1), 12–20. 10.1016/j.ab.2012.02.016 22370280

[B45] Kruspe S. Hahn U. (2014). An aptamer intrinsically comprising 5-fluoro-2'-deoxyuridine for targeted chemotherapy. Angew. Chem. Int. Ed. Engl. 53 (39), 10541–10544. 10.1002/anie.201405778 25145319

[B46] Ku T. H. Zhang T. Luo H. Yen T. M. Chen P. W. Han Y. (2015). Nucleic acid aptamers: An emerging tool for biotechnology and biomedical sensing. Sensors (Basel) 15 (7), 16281–16313. 10.3390/s150716281 26153774 PMC4541879

[B47] Kushwaha A. Takamura Y. Nishigaki K. Biyani M. (2019). Competitive non-SELEX for the selective and rapid enrichment of DNA aptamers and its use in electrochemical aptasensor. Sci. Rep. 9 (1), 6642. 10.1038/s41598-019-43187-6 31040350 PMC6491428

[B48] Kwon W. S. Rha S. Y. Choi Y. H. Lee J. O. Park K. H. Jung J. J. (2006). Ribonucleotide reductase M1 (RRM1) 2464G>A polymorphism shows an association with gemcitabine chemosensitivity in cancer cell lines. Pharmacogenet. Genomics 16 (6), 429–438. 10.1097/01.fpc.0000204999.29924.da 16708051

[B49] Lai W.-Y. Huang B. T. Wang J. W. Lin P. Y. Yang P. C. (2016). A novel PD-L1-targeting antagonistic DNA aptamer with antitumor effects. Mol. Ther. Nucleic Acids 5 (12), e397. 10.1038/mtna.2016.102 27959341

[B50] Lakhin A. V. Tarantul V. Z. Gening L. V. (2013). Aptamers: Problems, solutions and prospects. Acta naturae. 5 (4), 34–43. 10.32607/20758251-2013-5-4-34-43 24455181 PMC3890987

[B51] Lam W. Leung C. H. Bussom S. Cheng Y. C. (2007). The impact of hypoxic treatment on the expression of phosphoglycerate kinase and the cytotoxicity of troxacitabine and gemcitabine. Mol. Pharmacol. 72 (3), 536–544. 10.1124/mol.106.033472 17565005

[B52] Lee J.-H. Canny M. D. De Erkenez A. Krilleke D. Ng Y. S. Shima D. T. (2005). A therapeutic aptamer inhibits angiogenesis by specifically targeting the heparin binding domain of VEGF165. Proc. Natl. Acad. Sci. U. S. A. 102 (52), 18902–18907. 10.1073/pnas.0509069102 16357200 PMC1323181

[B53] Leigh Ann Anderson P. S. S. Kaci Durbin M. D. (2020). Sophia entringer, PharmD. Judith stewart, BPharm. Philip thornton, DipPharm. Carmen fookes, BPharm. Melisa puckey, BPharm. Gemcitabine. June 29, 2020; Available at:https://www.drugs.com/monograph/gemcitabine.html .

[B54] Li F. Lu J. Liu J. Liang C. Wang M. Wang L. (2017). A water-soluble nucleolin aptamer-paclitaxel conjugate for tumor-specific targeting in ovarian cancer. Nat. Commun. 8 (1), 1390. 10.1038/s41467-017-01565-6 29123088 PMC5680242

[B55] Li N. Nguyen H. H. Byrom M. Ellington A. D. (2011). Inhibition of cell proliferation by an anti-EGFR aptamer. PloS one 6 (6), e20299. 10.1371/journal.pone.0020299 21687663 PMC3110755

[B56] Li W. Wang S. Zhou L. Cheng Y. Fang J. (2019). An ssDNA aptamer selected by Cell-SELEX for the targeted imaging of poorly differentiated gastric cancer tissue. Talanta 199, 634–642. 10.1016/j.talanta.2019.03.016 30952308

[B57] Li Y. Li F. Jiang F. Lv X. Zhang R. Lu A. (2016). A mini-Review for cancer immunotherapy: Molecular Understanding of PD-1/PD-L1 pathway & translational Blockade of immune checkpoints . Int. J. Mol. Sci. 17 (7), E1151. 10.3390/ijms17071151 PMC4964524 27438833

[B58] Li Y. Liu J. (2020). Aptamer-based strategies for recognizing adenine, adenosine, ATP and related compounds. Analyst 145 (21), 6753–6768. 10.1039/d0an00886a 32909556

[B59] Liang C. Li F. Wang L. Zhang Z. K. Wang C. He B. (2017). Tumor cell-targeted delivery of CRISPR/Cas9 by aptamer-functionalized lipopolymer for therapeutic genome editing of VEGFA in osteosarcoma. Biomaterials 147, 68–85. 10.1016/j.biomaterials.2017.09.015 28938163

[B60] Lisi S. Fiore E. Scarano S. Pascale E. Boehman Y. Duconge F. (2018). Non-SELEX isolation of DNA aptamers for the homogeneous-phase fluorescence anisotropy sensing of tau Proteins. Anal. Chim. Acta 1038, 173–181. 10.1016/j.aca.2018.07.029 30278900

[B61] Liu C. Zheng J. Deng L. Ma C. Li J. Li Y. (2015). Targeted intracellular controlled drug delivery and tumor therapy through *in situ* forming Ag nanogates on mesoporous silica nanocontainers. ACS Appl. Mat. Interfaces 7 (22), 11930–11938. 10.1021/acsami.5b01787 25966745

[B62] Liu J. Stormo G. D. (2005). Combining SELEX with quantitative assays to rapidly obtain accurate models of protein-DNA interactions. Nucleic Acids Res. 33 (17), e141. 10.1093/nar/gni139 16186128 PMC1236725

[B63] Lu Y. Wu P. Yin Y. Zhang H. Cai C. (2014). Aptamer-functionalized graphene oxide for highly efficient loading and cancer cell-specific delivery of antitumor drug. J. Mat. Chem. B 2 (24), 3849–3859. 10.1039/c4tb00521j 32261731

[B64] Macugen (2011). Withdrawal of the application to change the marketing authorisation. [cited 2022 June 9]; Available at:https://www.ema.europa.eu/en/medicines/human/withdrawn-applications/macugen .

[B65] Mahajan U. M. Li Q. Alnatsha A. Maas J. Orth M. Maier S. H. (2021). Tumor-specific delivery of 5-fluorouracil-incorporated epidermal growth factor receptor-targeted aptamers as an efficient treatment in pancreatic ductal adenocarcinoma models. Gastroenterology 161 (3), 996–1010.e1. 10.1053/j.gastro.2021.05.055 34097885

[B66] Mahajan U. M. Li Q. Alnatsha A. Maas J. Orth M. Maier S. H. (2021). Tumor-specific delivery of 5-fluorouracil-incorporated epidermal growth factor receptor-targeted aptamers as an efficient treatment in pancreatic ductal adenocarcinoma models. Gastroenterology 161 (3), 996–1010.e1. 10.1053/j.gastro.2021.05.055 34097885

[B67] Mandal M. Dutta N. Dutta G. (2021). Aptamer-based biosensors and their implications in COVID-19 diagnosis. Anal. Methods 13 (45), 5400–5417. 10.1039/d1ay01519b 34751684

[B68] McKeague M. Derosa M. C. (2012). Challenges and opportunities for small molecule aptamer development. J. Nucleic Acids 2012, 748913. 10.1155/2012/748913 23150810 PMC3488411

[B69] Mendonsa S. D. Bowser M. T. (2004). *In vitro* selection of high-affinity DNA ligands for human IgE using capillary electrophoresis. Anal. Chem. 76 (18), 5387–5392. 10.1021/ac049857v 15362896

[B70] Mercier M.-C. Dontenwill M. Choulier L. (2017). Selection of nucleic acid aptamers targeting tumor cell-surface protein biomarkers. Cancers 9 (6), E69. 10.3390/cancers9060069 PMC5483888 28635657

[B71] Meyer C. Eydeler K. Magbanua E. Zivkovic T. Piganeau N. Lorenzen I. (2012). Interleukin-6 receptor specific RNA aptamers for cargo delivery into target cells. RNA Biol. 9 (1), 67–80. 10.4161/rna.9.1.18062 22258147 PMC3342945

[B72] Mongelard F. Bouvet P. (2010). AS-1411, a guanosine-rich oligonucleotide aptamer targeting nucleolin for the potential treatment of cancer, including acute myeloid leukemia. Curr. Opin. Mol. Ther. 12 (1), 107–114. 20140822

[B73] Moore A. Y. (2009). Clinical applications for topical 5-fluorouracil in the treatment of dermatological disorders. J. Dermatol. Treat. 20 (6), 328–335. 10.3109/09546630902789326 19954388

[B74] Motea E. A. Berdis A. J. (2010). Terminal deoxynucleotidyl transferase: The story of a misguided DNA polymerase. Biochim. Biophys. Acta 1804 (5), 1151–1166. 10.1016/j.bbapap.2009.06.030 19596089 PMC2846215

[B75] Nakatsura T. Yoshitake Y. Senju S. Monji M. Komori H. Motomura Y. (2003). Glypican-3, overexpressed specifically in human hepatocellular carcinoma, is a novel tumor marker. Biochem. Biophys. Res. Commun. 306 (1), 16–25. 10.1016/s0006-291x(03)00908-2 12788060

[B76] Ng E. W. M. Shima D. T. Calias P. Cunningham E. T. Guyer D. R. Adamis A. P. (2006). Pegaptanib, a targeted anti-VEGF aptamer for ocular vascular disease. Nat. Rev. Drug Discov. 5 (2), 123–132. 10.1038/nrd1955 16518379

[B77] Nucleoside Analogues (2012). “Nucleoside analogues,” in LiverTox: Clinical and research information on drug-induced liver injury (Bethesda (MD): National Institute of Diabetes and Digestive and Kidney Diseases). 31643176

[B78] Parashar A. Bhushan V. Mahanandia N. C. Kumar S. Mohanty A. K. (2022). Non-SELEX method for aptamer selection against β-casomorphin-7 peptide. J. Dairy Sci. 105, 5545–5560. 10.3168/jds.2021-21569 35534270

[B79] Park J. Y. Chae J. R. Cho Y. L. Kim Y. Lee D. Lee J. K. (2020). Targeted therapy of hepatocellular carcinoma using gemcitabine-incorporated GPC3 aptamer. Pharmaceutics 12 (10), E985. 10.3390/pharmaceutics12100985 PMC7588995 33080969

[B80] Park J. Y. Cho Y. L. Chae J. R. Moon S. H. Cho W. G. Choi Y. J. (2018). Gemcitabine-incorporated G-quadruplex aptamer for targeted drug delivery into pancreas cancer. Mol. Ther. Nucleic Acids 12, 543–553. 10.1016/j.omtn.2018.06.003 30195790 PMC6077122

[B81] Pharmacists T. A. S. o. H.-S. (2016). Fluorouracil topical.

[B82] Platella C. Riccardi C. Montesarchio D. Roviello G. N. Musumeci D. (2017). G-quadruplex-based aptamers against protein targets in therapy and diagnostics. Biochim. Biophys. Acta. Gen. Subj. 1861, 1429–1447. 10.1016/j.bbagen.2016.11.027 27865995 PMC7117017

[B83] Qi J. Zeng Z. Chen Z. Nipper C. Liu X. Wan Q. (2022). Aptamer-gemcitabine conjugates with enzymatically cleavable linker for targeted delivery and intracellular drug release in cancer cells. Pharm. (Basel, Switz. 15 (5), 558. 10.3390/ph15050558 PMC9147807 35631384

[B84] Ranches G. Lukasser M. Schramek H. Ploner A. Stasyk T. Mayer G. (2017). *In vitro* selection of cell-internalizing DNA aptamers in a model system of inflammatory kidney disease. Mol. Ther. Nucleic Acids 8, 198–210. 10.1016/j.omtn.2017.06.018 28918021 PMC5504087

[B85] Ray P. Cheek M. A. Sharaf M. L. Li N. Ellington A. D. Sullenger B. A. (2012). Aptamer-mediated delivery of chemotherapy to pancreatic cancer cells. Nucleic Acid. Ther. 22 (5), 295–305. 10.1089/nat.2012.0353 23030589 PMC3464421

[B86] Ray P. White R. R. (2010). Aptamers for targeted drug delivery. Pharm. (Basel) 3 (6), 1761–1778. 10.3390/ph3061761 PMC4033951 27713328

[B87] Ray P. White R. R. (2017). Cell-SELEX identifies a "sticky" RNA aptamer sequence. J. Nucleic Acids 2017, 4943072. 10.1155/2017/4943072 28194280 PMC5282457

[B88] Rosell R. Cobo M. Isla D. Camps C. Massuti B. (2006). Pharmacogenomics and gemcitabine. Ann. Oncol. 17, v13–v16. 10.1093/annonc/mdj942 16807441

[B89] Rosell R. Taron M. Sanchez J. M. Moran T. Reguart N. Besse B. (2004). The promise of pharmacogenomics: Gemcitabine and pemetrexed. Oncol. Willist. Park) 18, 70–76. 15655942

[B90] Rozenblum G. T. Lopez V. G. Vitullo A. D. Radrizzani M. (2016). Aptamers: Current challenges and future prospects. Expert Opin. Drug Discov. 11 (2), 127–135. 10.1517/17460441.2016.1126244 26630462

[B91] Russo Krauss I. Pica A. Merlino A. Mazzarella L. Sica F. (2013). Duplex-quadruplex motifs in a peculiar structural organization cooperatively contribute to thrombin binding of a DNA aptamer. Acta Crystallogr. D. Biol. Crystallogr. 69, 2403–2411. 10.1107/S0907444913022269 24311581

[B92] Sarac I. Hollenstein M. (2019). Terminal deoxynucleotidyl transferase in the synthesis and modification of nucleic acids. Chembiochem 20 (7), 860–871. 10.1002/cbic.201800658 30451377

[B93] Sathiyaseelan A. Saravanakumar K. Mariadoss A. V. A. Wang M. H. (2021). pH-controlled nucleolin targeted release of dual drug from chitosan-gold based aptamer functionalized nano drug delivery system for improved glioblastoma treatment. Carbohydr. Polym. 262, 117907. 10.1016/j.carbpol.2021.117907 33838795

[B95] Sefah K. Shangguan D. Xiong X. O'Donoghue M. B. Tan W. (2010). Development of DNA aptamers using Cell-SELEX. Nat. Protoc. 5 (6), 1169–1185. 10.1038/nprot.2010.66 20539292

[B96] Shamah S. M. Healy J. M. Cload S. T. (2008). Complex target SELEX. Acc. Chem. Res. 41 (1), 130–138. 10.1021/ar700142z 18193823

[B97] Shangguan D. Cao Z. Meng L. Mallikaratchy P. Sefah K. Wang H. (2008). Cell-specific aptamer probes for membrane protein elucidation in cancer cells. J. Proteome Res. 7 (5), 2133–2139. 10.1021/pr700894d 18363322 PMC2749249

[B98] Sheikh A. Md S. Alhakamy N. A. Kesharwani P. (2022). Recent development of aptamer conjugated chitosan nanoparticles as cancer therapeutics. Int. J. Pharm. 620, 121751. 10.1016/j.ijpharm.2022.121751 35436511

[B99] Shigdar S. Schrand B. Giangrande P. H. de Franciscis V. (2021). Aptamers: Cutting edge of cancer therapies. Mol. Ther. 29 (8), 2396–2411. 10.1016/j.ymthe.2021.06.010 34146729 PMC8353241

[B100] Sousa R. Padilla R. (1995). A mutant T7 RNA polymerase as a DNA polymerase. Embo J. 14 (18), 4609–4621. 10.1002/j.1460-2075.1995.tb00140.x 7556104 PMC394553

[B101] Stoker S. D. Novalic Z. Wildeman M. A. Huitema A. D. R. Verkuijlen S. A. W. M. Juwana H. (2015). Epstein-Barr virus-targeted therapy in nasopharyngeal carcinoma. J. Cancer Res. Clin. Oncol. 141 (10), 1845–1857. 10.1007/s00432-015-1969-3 25920375 PMC11823716

[B102] Sun H. Zu Y. (2015). A highlight of recent advances in aptamer technology and its application. Molecules 20 (7), 11959–11980. 10.3390/molecules200711959 26133761 PMC6331864

[B103] Sun K. Li J. (2022). A new method based on guanine rich aptamer structural change for carcinoembryonic antigen detection. Talanta 236, 122867. 10.1016/j.talanta.2021.122867 34635249

[B104] Sun Y. Gao F. Yang C. Li Y. Jin C. Xie S. (2020). Construction of bispecific aptamer-drug conjugate by a hybrid chemical and biological approach. Bioconjug. Chem. 31 (5), 1289–1294. 10.1021/acs.bioconjchem.0c00071 32223180

[B105] Sung H. Ferlay J. Siegel R. L. Laversanne M. Soerjomataram I. Jemal A. (2021). Global cancer statistics 2020: GLOBOCAN estimates of incidence and mortality worldwide for 36 cancers in 185 countries. Ca. Cancer J. Clin. 71 (3), 209–249. 10.3322/caac.21660 33538338

[B106] Thiel K. W. Hernandez L. I. Dassie J. P. Thiel W. H. Liu X. Stockdale K. R. (2012). Delivery of chemo-sensitizing siRNAs to HER2+-breast cancer cells using RNA aptamers. Nucleic Acids Res. 40 (13), 6319–6337. 10.1093/nar/gks294 22467215 PMC3401474

[B107] Tok J. Lai J. Leung T. Li S. F. Y. (2010). Selection of aptamers for signal transduction proteins by capillary electrophoresis. Electrophoresis 31 (12), 2055–2062. 10.1002/elps.200900543 20564698

[B108] Tong J. T. W. Harris P. W. R. Brimble M. A. Kavianinia I. (2021). An insight into FDA approved antibody-drug conjugates for cancer therapy. Molecules. 26. Basel, Switzerland), 5847. 10.3390/molecules26195847 34641391 PMC8510272

[B109] Tran B. T. Kim J. Ahn D. R. (2020). Systemic delivery of aptamer-drug conjugates for cancer therapy using enzymatically generated self-assembled DNA nanoparticles. Nanoscale 12 (45), 22945–22951. 10.1039/d0nr05652a 33188383

[B110] Tuerk C. Gold L. (1990). Systematic evolution of ligands by exponential enrichment: RNA ligands to bacteriophage T4 DNA polymerase. Science 249 (4968), 505–510. 10.1126/science.2200121 2200121

[B111] Ugrinova I. Petrova M. Chalabi-Dchar M. Bouvet P. (2018). Multifaceted nucleolin protein and its molecular partners in oncogenesis. Adv. Protein Chem. Struct. Biol. 111, 133–164. 10.1016/bs.apcsb.2017.08.001 29459030

[B112] Umar M. I. Chan C. Y. Kwok C. K. (2022). Development of RNA G-quadruplex (rG4)-targeting L-RNA aptamers by rG4-SELEX. Nat. Protoc. 17, 1385–1414. 10.1038/s41596-022-00679-6 35444329

[B113] Vernejoul F. Ghenassia L. Souque A. Lulka H. Drocourt D. Cordelier P. (2006). Gene therapy based on gemcitabine chemosensitization suppresses pancreatic tumor growth. Mol. Ther. 14 (6), 758–767. 10.1016/j.ymthe.2006.07.010 17000136

[B114] Wan L.-Y. Yuan W. F. Ai W. B. Ai Y. W. Wang J. J. Chu L. Y. (2019). An exploration of aptamer internalization mechanisms and their applications in drug delivery. Expert Opin. Drug Deliv. 16 (3), 207–218. 10.1080/17425247.2019.1575808 30691313

[B115] Weidle U. H. Maisel D. Klostermann S. Schiller C. Weiss E. H. (2011). Intracellular proteins displayed on the surface of tumor cells as targets for therapeutic intervention with antibody-related agents. Cancer Genomics Proteomics 8 (2), 49–63. 21471515

[B116] Wu X. Shaikh A. B. Yu Y. Li Y. Ni S. Lu A. (2017). Potential diagnostic and therapeutic applications of oligonucleotide aptamers in breast cancer. Int. J. Mol. Sci. 18 (9), E1851. 10.3390/ijms18091851 PMC5618500 28841163

[B117] Yamauchi N. Watanabe A. Hishinuma M. Ohashi K. I. Midorikawa Y. Morishita Y. (2005). The glypican 3 oncofetal protein is a promising diagnostic marker for hepatocellular carcinoma. Mod. Pathol. 18 (12), 1591–1598. 10.1038/modpathol.3800436 15920546

[B118] Yan J. Gao T. Lu Z. Yin J. Zhang Y. Pei R. (2021). Aptamer-targeted photodynamic platforms for tumor therapy. ACS Appl. Mat. Interfaces 13 (24), 27749–27773. 10.1021/acsami.1c06818 34110790

[B119] Yang G. H. Lee Y. B. Kang D. Choi E. Nam Y. Lee K. H. (2021). Overcome the barriers of the skin: Exosome therapy. Biomater. Res. 25 (1), 22. 10.1186/s40824-021-00224-8 34217362 PMC8254055

[B120] Yang X. Li N. Gorenstein D. G. (2011). Strategies for the discovery of therapeutic aptamers. Expert Opin. Drug Discov. 6 (1), 75–87. 10.1517/17460441.2011.537321 21359096 PMC3045091

[B121] Yazdian-Robati R. Bayat P. Oroojalian F. Zargari M. Ramezani M. Taghdisi S. M. (2020). Therapeutic applications of AS1411 aptamer, an update review. Int. J. Biol. Macromol. 155, 1420–1431. 10.1016/j.ijbiomac.2019.11.118 31734366

[B122] Yoon S. Huang K. W. Reebye V. Mintz P. Tien Y. W. Lai H. S. (2016). Targeted delivery of C/EBPα -saRNA by pancreatic ductal adenocarcinoma-specific RNA aptamers inhibits tumor growth *in vivo* . Mol. Ther. 24 (6), 1106–1116. 10.1038/mt.2016.60 26983359 PMC4923325

[B123] Yoon S. Huang K. W. Reebye V. Spalding D. Przytycka T. M. Wang Y. (2017). Aptamer-drug conjugates of active metabolites of nucleoside analogs and cytotoxic agents inhibit pancreatic tumor cell growth. Mol. Ther. Nucleic Acids 6, 80–88. 10.1016/j.omtn.2016.11.008 28325302 PMC5363417

[B124] Yoon S. Huang K. W. Reebye V. Spalding D. Przytycka T. M. Wang Y. (2017). Aptamer-drug conjugates of active metabolites of nucleoside analogs and cytotoxic agents inhibit pancreatic tumor cell growth. Mol. Ther. Nucleic Acids 6, 80–88. 10.1016/j.omtn.2016.11.008 28325302 PMC5363417

[B125] Yoon S. Rossi J. J. (2018). Aptamers: Uptake mechanisms and intracellular applications. Adv. Drug Deliv. Rev. 134, 22–35. 10.1016/j.addr.2018.07.003 29981799 PMC7126894

[B126] Yu X. Yu Y. (2014). A mathematical analysis of the selective enrichment of NECEEM-based non-SELEX. Appl. Biochem. Biotechnol. 173 (8), 2019–2027. 10.1007/s12010-014-0989-9 24861320

[B127] Zhan Y. Ma W. Zhang Y. Mao C. Shao X. Xie X. (2019). DNA-based nanomedicine with targeting and enhancement of therapeutic efficacy of breast cancer cells. ACS Appl. Mat. Interfaces 11 (17), 15354–15365. 10.1021/acsami.9b03449 30924334

[B128] Zhang Y. Juhas M. Kwok C. K. (2022). Aptamers targeting SARS-COV-2: A promising tool to fight against COVID-19. Trends Biotechnol. 10.1016/j.tibtech.2022.07.012 PMC9340053 35995601

[B129] Zhang Y. Xie X. Yeganeh P. N. Lee D. J. Valle-Garcia D. Meza-Sosa K. F. (2021). Immunotherapy for breast cancer using EpCAM aptamer tumor-targeted gene knockdown. Proc. Natl. Acad. Sci. U. S. A. 118 (9), e2022830118. 10.1073/pnas.2022830118 33627408 PMC7936362

[B130] Zhao Z. Xu L. Shi X. Tan W. Fang X. Shangguan D. (2009). Recognition of subtype non-small cell lung cancer by DNA aptamers selected from living cells. Analyst 134 (9), 1808–1814. 10.1039/b904476k 19684903

[B131] Zhong J. Ding J. Deng L. Xiang Y. Liu D. Zhang Y. (2021). Selection of DNA aptamers recognizing EpCAM-positive prostate cancer by cell-SELEX for *in vitro* and *in vivo* MR imaging. Drug Des. devel. Ther. 15, 3985–3996. 10.2147/DDDT.S322854 PMC8464308 34584404

[B132] Zhu L. Yang J. Ma Y. Zhu X. Zhang C. (2022). Aptamers entirely built from therapeutic nucleoside analogues for targeted cancer therapy. J. Am. Chem. Soc. 144 (4), 1493–1497. 10.1021/jacs.1c09574 35073490

[B133] Zhu Z. Song Y. Li C. Zou Y. Zhu L. An Y. (2014). Monoclonal surface display SELEX for simple, rapid, efficient, and cost-effective aptamer enrichment and identification. Anal. Chem. 86 (12), 5881–5888. 10.1021/ac501423g 24863283

